# Species-specific mutual regulation of p53 and miR-138 between human, rat and mouse

**DOI:** 10.1038/srep26187

**Published:** 2016-05-17

**Authors:** Jie Li, Wei Xia, Xueting Su, Xingliang Qin, Ying Chen, Shaohua Li, Jie Dong, Hongmei Ding, Hui Li, Aixue Huang, Xingfeng Ge, Lvbin Hou, Chaonan Wang, Leqiao Sun, Chenjun Bai, Xuelian Shen, Tao Fang, Yuanlin Liu, Yi Zhang, Hongru Zhang, Hongwen Zhang, Ningsheng Shao

**Affiliations:** 1 Department of Biochemistry and Molecular Biology, Institute of Basic Medical Sciences, Beijing 100850, China; 2 Department of Cell Biology, Institute of Basic Medical Sciences, Beijing 100850, China; 3 Otorhinolaryngological department, Haidian Section of Peking University third Hospital, Beijing 100080, China; 4 Department of Interventional Radiology, General Hospital of Fuzhou, Fuzhou, China

## Abstract

In recent years, p53 was identified to regulate the expression of many miRNAs and was also regulated by miRNAs. In this paper, we found that miR-138 showed a pronounced increase after p53 activation in human non-small cell lung cancer (NSCLC) cells, which is mediated by p53 binding sites in the promoter region of its host gene, but this did not happen with rat and mouse cells. More interestingly, we found that p53 could be also regulated by miR-138 in mouse and rat cells, but not in the human NSCLC cells. Our results suggest the existence of species-specific differences of the regulations of miRNA against its targets and the regulations of miRNA itself by other proteins.

Tumour protein p53 is crucial in multicellular organisms and functions as a tumour suppressor. Since p53 was discovered 35 years ago, a significant amount of research has been performed on this single protein, its signalling pathway and its complex network^1^. As a transcription factor, the activated p53 protein binds to a specific DNA sequence, termed the p53-responsive element (RE) to regulate p53 target genes. A p53-RE is composed of RRRCWWGYYY (spacer of 0–21 nucleotides) RRRCWWGYYY, where R is a purine, W is A or T, and Y is a pyrimidine^2^. P53 directly activates a large set of genes, which mediate numerous cellular functions that contribute to tumour suppression.

MicroRNAs are a class of evolutionarily conserved, non-coding, short RNAs comprising approximately 22 nucleotides, which reduce mRNA stability or inhibit translation by complementary base pairing to the 3′ untranslated region (3′ UTR) of their target genes. P53 regulates the expression of many miRNAs, such as the miR-34 family, miR-200 family, miR-192 family, miR-107, miR-145, miR-15a and miR-16-1^3^,^4^,^5^,^6^,^7^,^8^,^9^,^10^. P53 is also regulated by miRNAs such as miR-125b, miR-504, miR-33, miR-380-5p, miR-1285, miR-200a, miR-30a/b/d and miR-25^11^,^12^,^13^,^14^,^15^,^16^,^17^. The mutual regulation of p53-miRNAs suggests the existence of a “ transcription factor – miRNA feedback loop” which would be important in gene regulation^18^.

MiR-138 as a potential tumour suppressor has various biological functions, including roles in tumor progression and metastasis, cell differentiation, DNA damage and disease^19^,^20^,^21^. The expression of miR-138 is generally low in tumours such as thyroid cancer, lung cancer, leukemia, neck squamous cell carcinomas, tongue squamous cell carcinoma, nasopharyngeal carcinoma, gallbladder carcinoma, pancreatic carcinoma and cervical cancer^22^,^23^,^24^,^25^,^26^,^27^,^28^. It was previously reported that HeLa cells with low level of miR-138 may contain a factor that specifically recognizes miR-138 precursor and inhibits its processing by Dicer. However, such inhibition factors have not been identified until now since 2006. We suspect that the regulation of miR-138 biogenesis may not only happen at a post- transcriptional level for miR-138 low expression. By coincidence, HeLa cell line is a HPV-18-positive human cervical carcinoma cell line with the high instability of p53 proteins^29^.Whether is there a connection between the low activity of p53 and low level of miR-138? On the other hand, Ye *et al*. reported that miR-138 directly targeted the 3′ untranslated region (UTR) of p53^30^. So, is there a feedback loop between p53 and miR-138?

To explore the relation of p53 to miR-138, the human non-small cell lung cancer cell lines (NSCLC) H460 with p53 wild-type background, H1299 with p53-null background and other mouse and rat cells were used for comparison in this work. This leads to an unexpected finding that the regulation between p53 and miR-138 is unidirectional with no feedback in human NSCLC cells. More interestingly, the regulation between p53 and miR-138 is different between human, mouse and rat cells.

## Results

### P53-mediated activation of miR-138 in human NSCLC cells

**Figure 1:**
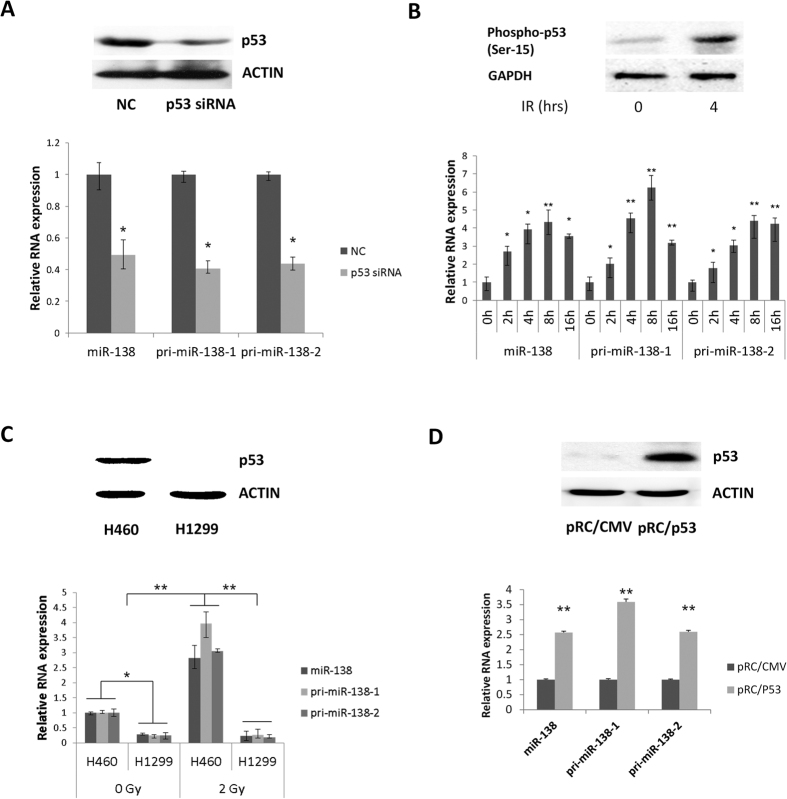
P53 regulates miR-138 in human non-small cell lung cancer cells. (**A**) Western blot analyses were performed to examine the effect of p53 siRNA on endogenous p53 protein levels in H460 cells (top); qRT-PCR assays were used to examine miR-138 and pri-miR-138 levels in H460 cells treated with p53 siRNA for 48 h (bottom). (**B**) Western blot analyses were performed to examine phosphorylated p53 protein levels in H460 cells treated with 2 Gy of ionizing radiation for the indicated times (0 and 4 hours) (top). Quantitative RT-PCR assays were performed to examine miR-138, pri-miR-138-1 and pri-miR-138-2 levels in H460 cells treated with 2 Gy of ionizing radiation for the indicated times (0, 2, 4, 8 and 16 hours) (bottom). U6 snRNA served as an internal control for miRNAs; GAPDH mRNA was used for normalization of pri-miRNA levels. (**C**) Western blot analyses were performed to examine p53 protein levels in H460 and H1299 cells (top); quantitative RT-PCR assays were performed to examine miR-138, pri-miR-138-1 and pri-miR-138-2 levels in H460 and H1299 cells treated with different doses (0 and 2 Gy) of ionizing radiation for the indicated time (4 hour) (bottom). (**D**) Western blot analyses were performed to examine p53 protein levels in H1299 cells transfected with pRC/CMV or pRC/p53 plasmids for 48h (top). Quantitative RT-PCR assays were performed to examine miR-138, pri-miR-138-1 and pri-miR-138-2 levels in H1299 cells transfected with pRC/CMV or pRC/p53 plasmids for 48h (bottom). Results are expressed as means ± s.d. for three independent experiments. *P < 0.05; **P < 0.01 versus control.

The human miR-138 family comprises hsa-miR-138-1 and hsa-miR-138-2, located on chromosomes 3p21.32 and 16q13, respectively. To explore the relation of p53 to miR-138, we firstly silenced p53 expression in human lung cancer H460 cells (p53 wild-type cells) using a p53 siRNA, and observed that the expression levels of miR-138 and its precursors were significantly downregulated in H460 cells (Fig. 1A). We then treated H460 cells with 2 Gy of ionizing radiation, which promotes the phosphorylation and transcriptional regulatory activity of p53^31^. Under these conditions, the mature level and the primary level of miR-138 were significantly upregulated (Fig. 1B). To further determine the regulations of miR-138 by p53 in human lung cancer cells, we compared the expressions of p53 and miR-138 in H460 and H1299 cells, which lack p53 expression. The expression level of miR-138 was significantly higher in H460 cells than in H1299 cells under both the natural state and under stimulation by ionizing radiation (Fig. 1C). Correspondingly, the expression levels of mature miR-138 and its precursors increased significantly after overexpression of wild-type p53 in H1299 cells (Fig. 1D).

### Characterization of p53 binding elements in the miR-138 gene

**Figure 2:**
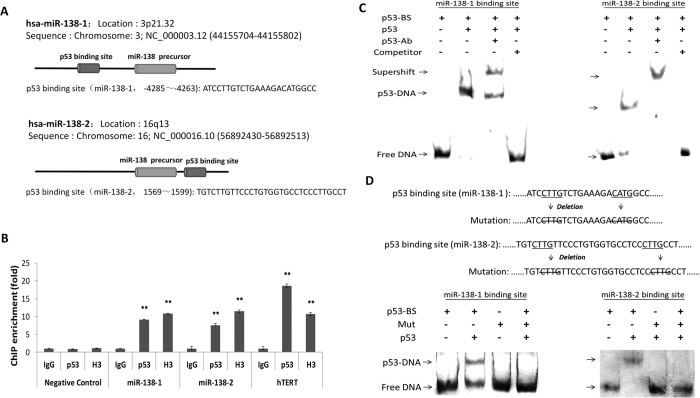
p53 binds to the predicted p53 binding site of hsa-miR-138. (**A**) Schematic illustration showing that pre-miR-138-1 and pre-miR-138-2 are both intergenic. A putative p53 binding site (BS) located −4285 to −4263 bp upstream of miR-138-1; a predicted p53 binding site located downstream (1569 to 1599 bp) of miR-138-2. (**B**) ChIP-qPCR analyses were preformed using digested chromatin from H460 cells. The predicted p53 binding region of miR-138 was amplified from immunoprecipitates of anti-p53, anti-Histone H3 or normal rabbit IgG control. The negative control primer for a “promoter free” region in the genome to monitor the background noise; hTERT primer was used as a positive control. The fold enrichment over the IgG control is represented (mean ± s.d.; n = 3; **P < 0.01). (**C**) p53 binds to the predicted binding site (BS) for miR-138 *in vitro*. Electrophoretic mobility shift assay (EMSA) was performed with 100 ng p53 wt protein and biotin-labelled oligonucleotides. The p53 antibody and unlabeled competitor probe were added as indicated. (**D**) The mutant p53-BS probes (top) were used in the EMSA with the p53 wt protein (bottom).

To elucidate whether p53 directly regulates the transcription of human miR-138 as a transcription factor, we searched for the potential binding sites of p53 in the 5 kb upstream and downstream genomic regions of the miR-138 transcript using p53MH algorithm^32^ and BioSun software^33^. Two relatively conserved p53 binding sites were found, one at upstream of the hsa-miR-138-1 transcription start site (−4263~−4285) and the other downstream of the start site (1569~1599) (Fig. 2A). We then performed ChIP (chromatin immunoprecipitation) analysis to investigate whether there is an interaction between p53 and these predicted binding sites. The results showed that the predicted binding sites could interact with p53 (Fig. 2B). Electrophoretic mobility shift assay (EMSA) experiments using wild-type p53 protein and biotin-labelled double-stranded DNA fragments representing the binding sites demonstrated the direct binding of p53 to the predicted binding sites (Fig. 2C). The corresponding deletion mutation of the “CWWG” (W=A or T) bases in these two predicted sites ablated wild-type p53 binding (Fig. 2D).

**Figure 3:**
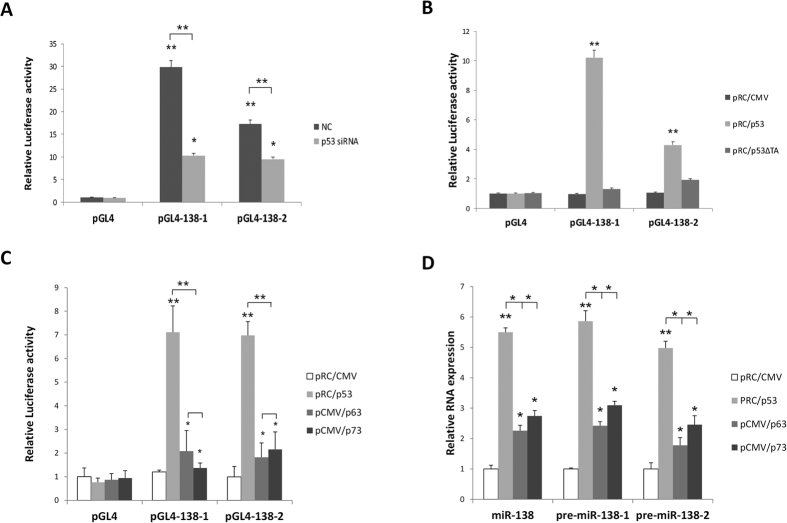
miR-138 is a transcriptional target of p53. (**A**) H460 (p53+/+) cells were co-transfected with luciferase reporter plasmids pGL4-138-1, pGL4-138-2 or empty pGL4-basic vector, with p53 siRNA or a control siRNA. After 48 h, the luciferase activity was measured. (**B**) H1299 (p53−/−) cells were co-transfected with luciferase reporter plasmids pGL4-138-1, pGL4-138-2 or empty pGL4-basic vector with pRC/p53, pRC/p53ΔTA or empty pRC/CMV plasmid. After 48 h, the luciferase activity was measured. (**C**) H1299 (p53−/−) cells were co-transfected with luciferase reporter plasmids pGL4-138-1, pGL4-138-2 or empty pGL4-basic vector with pRC/p53, pCMV/p63, pCMV/p73 or control pRC/CMV plasmid. After 48 h, the luciferase activity was measured. (**D**) H1299 (p53−/−) cells were transfected with pRC/p53, pCMV/p63, pCMV/p73 or control pRC/CMV plasmid. After 48 h, quantitative RT-PCR was performed to examine miR-138 and pri-miR-138 levels. Data are representative of at least three independent experiments (means ± s.d.). *P < 0.05; **P < 0.01 versus control.

To further detect whether p53 performs transcriptional activation after binding to the p53 binding sites, we incorporated them separately into the basic promoter of the firefly luciferase reporter gene (named as pGL4-138-1 and pGL4-138-2). As shown in (Fig. 3A), vectors containing the p53 binding sites exhibited significantly higher levels of luciferase activity in the presence of p53. When the expression level of p53 was downregulated by p53 siRNA, luciferase activity was also significantly inhibited. The empty vectors lacking the p53 binding sites showed low levels of luciferase activity, regardless of the presence or absence of p53. We then overexpressed wild-type p53 artificially in H1299 cells and co-transfected with the reporter vector containing p53 binding sites: high levels of luciferase activity were observed. When a mutant p53 lacking the transcriptional activation domain was overexpressed, no enhancement of the luciferase activity was observed (Fig. 3B). Taken together, these results indicated that p53 acts as a transcriptional activator after interaction with the binding sites in the miR-138 gene. Previous studies have shown that p53, p63 and p73 proteins have similar transcriptional activation and DNA binding abilities toward p53 responsive genes. To test whether p63 and p73 have transcriptional activation functions for these two binding sites in human miR-138 genes, we overexpressed the wild-type p63 and p73 in H1299 cells and co-transfected them with the reporter vector containing p53 binding sites. The results indicated that p63 and p73 could activate the luciferase activity to varying degrees, but not to the levels produced by p53 (Fig. 3C). Similarly, the transcriptional regulation of p53 on miR-138 was significantly stronger than that of p63 and p73 in H1299 cells (Fig. 3D). These results further confirmed the existence of p53 REs in the upstream and downstream of the has-miR-138 gene and that p53 transcriptionally activates miR-138 by binding to these sites.

### Mutual regulation differences of p53 - miR-138 in human, mouse and rat

**Figure 4:**
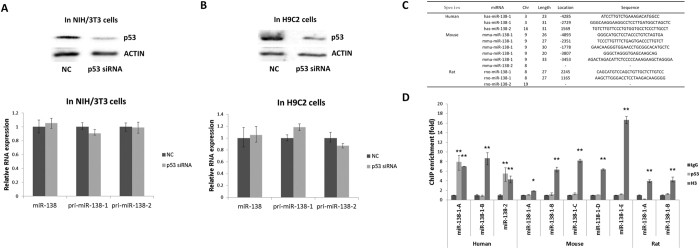
P53 regulation of miR-138 shows divergence between species. (A,B) Western blot analyses were performed to examine the effects of p53 siRNA on endogenous p53 protein levels in NIH/3T3 and H9C2 cells (top); quantitative RT-PCR assays were performed to examine miR-138 and pri-miR-138 levels in two cell types (NIH/3T3 and H9C2) treated with p53 siRNA for 48 h. (**C**) The putative p53 binding sites loacted upstream or downstream 5 kb of miR-138-1 or miR-138-2 in human, mouse and rat. (**D**) ChIP-qPCR analysis were performed using digested chromatin from H460, NIH/3T3 and H9C2 cells. The predicted p53 binding region of miR-138 was amplified from immunoprecipitates of anti-p53, anti-Histone H3 or normal rabbit IgG control.

To test the regulation of p53 on miR-138 in other species, we silenced the p53 expression in mouse NIH/3T3 cells and rat H9C2 cells (both p53 wild-type cells) using p53 siRNA. Surprisingly, the expression levels of miR-138 and its precursors showed no significant changes in these cells, which suggested that p53 regulation of miR-138 is species specific (Fig. 4A,B and Supplementary Fig. S3). Further analysis using Vista software (http://genome.lbl.gov/vista/mvista/submit.shtml) of the 5 kb upstream and downstream genomic regions of the miR-138 gene in human, mouse and rat showed little homology between humans and mice or rats (Supplementary Fig. S1). ChIP analysis failed to identify p53 binding sites in the mouse and rat miR-138 gene region, which explains the lack of response of miR-138 transcription to p53 in mouse and rat cells (Fig. 4C,D).

**Figure 5:**
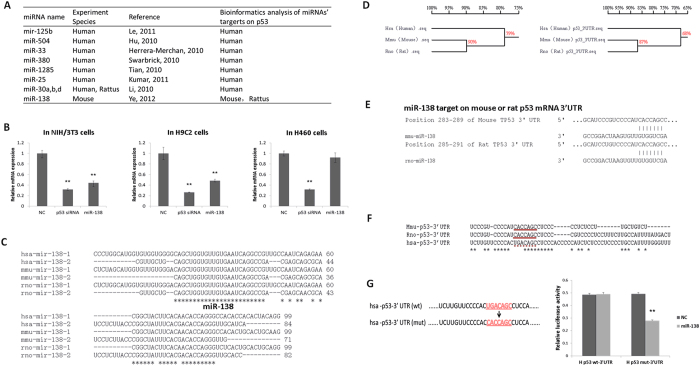
MiR-138 targeting p53 shows divergence between species. (**A**) Summary of the reported miRNAs targeting p53. (**B**) qRT-PCR assays were performed to examine the effects of miR-138 mimic transfection on p53 gene transcription (GAPDH served as an internal control). (**C**) Alignment of the miR-138 sequence. MiR-138 sequences from three species of mammals (Human, Mouse and Rat) were compared using ClustalW server (http://www.ebi.ac.uk/Tools/msa/clustalw2/). (**D**) Alignment of p53 amino acid sequences and p53 3′ UTR nucleotide sequences. (**E**) The predicted miR-138 targeting sequence located in the 3′ UTR of the p53 mRNA of rat and mouse. (**F**) Human p53 mRNA 3′ UTR lacks miR-138 targeting sequence, with a three-base difference. (**G**) Human p53 mRNA 3′ UTR (NM_001126114) was mutated to contain an miR-138 targeting sequence (left); dual luciferase reporter assays were performed to test the interaction of miR-138 with the wild-type predicted p53 3′ UTR targeting sequences (H p53 wt-3′ UTR) and the mutated targeting sequences (H p53-mut-3′ UTR). *P < 0.05 versus control. Data are representative of at least three independent experiments (means ± SD).

**Figure 6:**
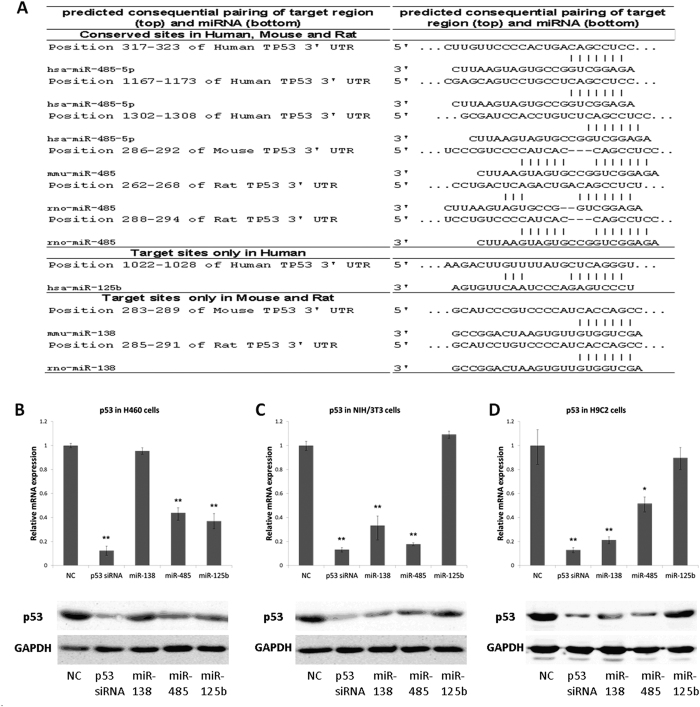
MiR-125b targeting p53 shows divergence between species. (**A**) The predicted miRNAs targeting sequences located in the 3′ UTR of the p53 mRNA of human, rat and mouse. The 3′UTR of human, mouse and rat p53 mRNA all have miR-485 conserved predicted target sites, but only the 3′UTRs of human p53 mRNA has miR-125b target site, (**B–D**) Quantitative RT-PCR assays were performed to examine p53 mRNA levels in three cell types (H460, NIH/3T3 and H9C2) treated with p53 siRNA and other miRNAs for 48 h. GAPDH served as the internal control (top). Western blot analyses were performed to examine the effects of p53 siRNA and miRNAs on endogenous p53 protein levels in H460, NIH/3T3 and H9C2 cells (bottom).

Surprisingly, when we reviewed previous report concerning the regulation of p53 by miR-138^30^, we found that the experiments were done only in mouse embryonic fibroblast cells and the majority of reports on the regulation of p53 by miRNAs were done in human cells (Fig. 5A). To elucidate the effect of miR-138 on p53 in humans, we overexpressed miR-138 in p53 wild-type human H460 cells found no downregulation of the p53 mRNA expression levels (Fig. 5B). To further elucidate the differences in p53 regulation by miR-138 among mouse, rat and human, we performed further bioinformatic analysis of the target sites for miR-138 in p53. First, we analysed the conservation of miR-138, which showed that miR-138 is highly conserved in many vertebrates. Although two miR-138 precursor molecules (pre-miR-138-1 and pre-miR-138-2) exist in human, mouse and rat, distributed on different chromosomes, the sequences of mature miR-138 were completely consistent in human, mouse and rat (Fig. 5C). However, we found the sequence of the p53 mRNA is relatively poorly conserved: the similarities of p53 mRNA coding regions and 3′ UTR regions of rats and mice are 79% and 68% similar, respectively, compared with that of human p53 (Fig. 5D and Supplementary Fig. S2). Further analysis of the possible target sites of miR-138 in the 3′ UTRs of mouse, rat and human p53 mRNA using TargetScan (http://www.targetscan.org), miRanda (http://www.microrna.org) and PicTar (http://www.pictar.org) in combination revealed that the 3′ UTRs of mouse and rat p53 mRNA have miR-138 target sites, but the 3′ UTR of human p53 mRNA has lost the miR-138 target sites because of mutations (Fig. 5E,F). We then confirmed the bioinformatics analysis experimentally. Overexpression of miR-138 in human H460 cells could not knock down p53 mRNA expression, while the p53 mRNA level decreased significantly in NIH/3T3 and H9C2 cells (Fig. 5B). Nevertheless, when we mutated the miR-138 binding site in the 3′ UTR of human p53 mRNA, we found that miR-138 could obviously interact with the mutated binding and reduce the activity of the luciferase reporter (Fig. 5G). In fact, not only miR-138, miR-125b regulation of p53 also has species specificity, compared with miR-485 without this feature. However, unlike miR-138, miR-125b specifically target human p53 instead of mouse and rat (Fig. 6A,B).

**Figure 7:**
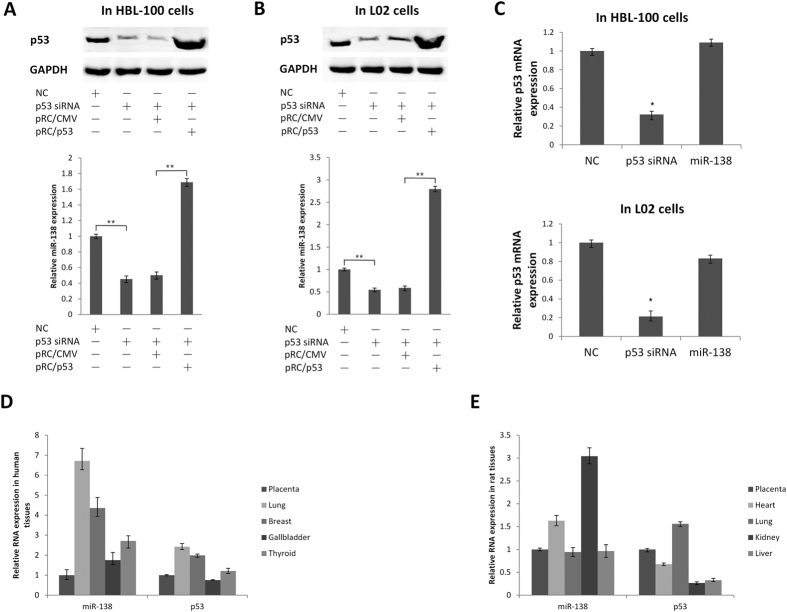
The relation between p53 and miR-138 in non-cancerous human cell lines and tissues. (**A,B**) Two normal cell lines (human normal breast cell HBL-100 and normal liver cell L02) were transfected with control or p53 siRNA. After treament for 24 h, cells were transfected with pRC/CMV or pRC/P53 plasmids (4 μg/well in 6-well plates) for another 48 h. Western blot analyses were performed to examine p53 protein levels (top); quantitative RT-PCR assays were performed to examine miR-138 and p53 levels (bottom). (**C**) Quantitative RT-PCR assays were performed to examine the effects of miR-138 mimic transfection on p53 gene transcription in HBL-100 and L02 cells (GAPDH served as an internal control). (**D,E**) Quantitative RT-PCR assays were performed to examine miR-138 and p53 relative levels in human and rat tissues.

To rule out tissue and cell type-specific regulation differences, we further chosed human normal cell lines instead of other cancerous cell lines to test the regulation between p53 and miR-138.We found that miR-138 decreased significantly after p53 “knock down” and restored when p53 overexpression in human normal breast cell HBL-100 and normal liver cell L02 (Fig. 7A,B). Conversely, p53 was not downregulated by miR-138 (Fig. 7C). That is, similar relation between p53 and miR-138 was found both in non-cancerous human cell lines and in NSCLC cell lines. We further compared the relation between p53 and miR-138 in a series of human and rat tissues, and found a nearly positive expression correlation between miR-138 and p53 in human tissues (placenta, lung, breast, gallbladder and thyroid) while a negative expression correlation in rat tissues (placenta, heart, lung, kidney and liver) (Fig. 7D,E). These results could confirm the existence of species-specific differences of the regulations between miR-138 and p53.

## Discussion

In this study, we found that p53 could regulate miR-138 and was regulated by miR-138, and the mutual regulation between p53 and miR-138 was confirmed as species-specific; i.e., the regulatory effects between p53 and miR-138 are unidirectional in mouse and human cells, which would lead to different biological effects on the molecules downstream of p53 and miR-138. We do not understand the exact implication of this unidirectional regulation; however, our results suggested that it might be associated with species evolution. It is not surprising that the expression patterns and regulation modes of coding genes and noncoding RNAs may have differences in different species, although a growing number of studies have found that mice have basically identical genes to humans. Previous studies have shown that miRNAs are highly evolutionarily conserved among different species; however, the cross-species differences of the regulations of miRNA against targets and the regulations to miRNA itself are still poorly understood. Thus, researchers should be very careful in selecting animal models in future miRNA studies.

Considering that miR-138 regulates p53 only in mouse and rat cells and that miR-138 could be regulated by p53 only in human cells, suggested that it is difficult to form the “ transcription factor – miRNA feedback loop” with same miRNA in the same cells, which may be important in stable gene regulation.

## Materials and Methods

### Cell culture and transfection and treatment

The human NSCLC cell lines H460 and H1299 were cultured in RPMI-1640 Medium (Sigma-Aldrich, Poole, UK), supplemented with 10% fetal bovine serum. The mouse embryo fibroblast cell line NIH/3T3 was cultured in Dulbecco’s Modified Eagle’s Medium with 10% bovine calf serum. The rat embryo fibroblast cell line H9C2, the human breast epithelial cell line HBL-100 and human liver cell line L02 were maintained in Dulbecco’s Modified Eagle’s Medium containing 10% fetal bovine serum. H460 and H1299 cells were obtained from Prof. Guo Ning (Department of Pathophysiology, Institute of Basic Medical Sciences, Beijing, China). The other cell lines were obtained from the Cell Resource Center of the Institute of Basic Medical Sciences (Beijing, China). Cells at 70% confluence were transfected with vectors, 20 nM siRNAs or 20 nM miRNA using Lipofectamine 2000 (Invitrogen, Carlsbad, CA, USA) according to manufacturer’s recommendations. Transcription inhibitor was added 24 hours after transcription by adding media containing Actinomycin D (10 μg/ml in DMSO).

The miRNA mimic and siRNA sequences were as follows: miR-138 mimic, 5′-AGCUGGUGUUGUGAAUCAGGCCG-3′ (sense); p53 siRNA, 5′-UAUGAAUCGUCGUCCUAUUC-3′ (sense).

### Western blot analysis

Whole-cell lysates were obtained using the ProteoJET™ Mammalian Cell Lysis Reagent (Thermo Scientific, Rockford, IL, USA). Proteins (50 μg) were separated by 10% SDS-PAGE and transferred to nitrocellulose membranes. The membrane was blocked with 5% non-fat dried milk in TBST (50 mM Tris [pH 7.5], 200 mM NaCl, 0.05% Tween 20) at room temperature for 1 h. The membrane was then incubated with primary antibody in the same concentration of milk in TBST for 1 h at room temperature, washed three times with TBST for 15 min, and then incubated with the HRP-conjugated secondary antibody at room temperature for 1 h. The membrane was again washed three times with TBST. Antigen-antibody complexes were detected using the SuperSignal detection reagents (Thermo Scientific, Rockford, IL, USA). The following antibodies were used: rabbit monoclonal anti-p53, rabbit polyclonal anti-Phospho-p53, mouse polyclonal anti-GAPDH, mouse polyclonal anti-ACTIN (Cell Signaling Inc. Danvers, MA, USA); secondary antibodies (goat-anti-rabbit and goat-anti-mouse) conjugated to horseradish peroxidase (Santa Cruz. CA, USA).

### ChIP assay

The ChIP assay was performed using the SimpleChIP® Plus Enzymatic Chromatin IP Kit (Cell Signaling Inc.) according to the manufacturer’s protocol. Immunoprecipitation was carried out using the mouse monoclonal p53 antibody. A histone H3 monoclonal antibody was used as a positive control and unrelated rabbit IgG was the negative control. To amplify the potential p53-binding sites from the upstream or downstream genomic regions of miR-138, real-time PCR was performed. The negative control primer for a “promoter free” region in the genome was used to monitor the background noise. The human telomerase (hTERT) primer was used as a positive control for wild-type p53 binding. Primer sequences used for ChIP are listed in Table S1.

### EMSA

Recombinant p53 protein was purchased from OriGene (OriGene Technologies, Rockville, MD, USA). The double-stranded oligonucleotides harbouring the p53 binding sequence (miR-138-1 sense: 5′-ATCCTTGTCTGAAAGACATGGCC-3′; miR-138-1 antisense: 5′-GGCCATGTCTTTCAGACAAGGAT-3′; miR-138-2 sense: 5′-TGTCTTGTTCCCTGTGGTGCCTCCCTTGCCT-3′, miR-138-2 antisense: 5′-AGGCAAGGGAGGCACCACAGGGAACAAGACA-3′) were end labelled by biotin (Invitrogen, Carlsbad, CA, USA). EMSAs were performed using the LightShift chemiluminescent EMSA kit (Thermo Scientific, Rockford, IL, USA) according to manufacturer’s recommendations. The labelled probes (20 fmol) were incubated with the p53 protein (200 ng) for 30 min in binding buffer in the presence or absence of unlabelled probes (1 pmol). If an antibody was added to detect the supershift, the antibody and protein were pre-incubated for 20 min before the labelled probes were added. All EMSA experiments were performed on 5% polyacrylamide gels in Tris-borate-EDTA buffer (45 mM Trisborate/1 mM EDTA). EMSA gels were electro-blotted onto Hybond-N+ membranes (GE Healthcare, Little Chalfont, Buckinghamshire, UK). Membranes were exposed to Clinx ChemiScope with a CCD camera (Clinx Science Instruments, Shanghai, China) for luminescence detection.

### Plasmids

Protein expression plasmids: pRC/CMV was used as a negative control (Promega, Madison, WI, USA); pRC/p53 containing the entire p53 coding region and pRC/p53ΔTA lacking the N-terminal first transactivation domain were described previously and were obtained from Sino Biological (Sino Biological Inc, Beijing, China). pCMV/p63 and pCMV/p73 were all purchased from OriGene (OriGene Technologies, Rockville, MD, USA).

Reporter plasmids for the transcriptional activation assay: pGL4.26[luc2/minP/Hygro] (pGL4 for short) containing a multiple cloning region for the insertion of a response element of interest upstream of a minimal promoter and the luc2 gene, was designed for high expression and reduced anomalous transcription (Promega). The p53-binding sites oligonucleotides (bold) contains *Xho*I at the 5′-end and *Hind*III sequence (italic) at the 3′-end (miR-138-1: 5′-*CTCGAG***ATCCTTGTCTGAAAGACATGGCC***AAGCTT*-3′; miR-138-2: 5′-*CTCGAG***TGTCTTGTTCCCTGTGGTGCCTCCCTTGCCT***AAGCTT*-3′) were separately inserted upstream of a minimal promoter of the luc2 gene and named pGL4-138-1 and pGL4-138-2.

Reporter plasmids for the miRNA target assay: The almost full length human *P53* 3′ UTR (1496 nt, GenBank accession NM_001126114) was PCR amplified using genomic DNA from H460 cells. The final PCR product was cloned into the pGL3-Control Vector (Promega between *Mlu*I and *Bgl*II restriction enzyme sites located downstream of the firefly luciferase reporter gene and named H p53 wt-3′UTR. The mutated human *P53* 3′ UTR containing a predicted miR-138 binding site was cloned into pGL3-Control Vector and named H p53 mut-3′UTR. All the miRNA target binding sites were mutated using the MutanBEST mutation kit (Takara, Tokyo, Japan). Primers for cloning (bold italic for restriction sites):

H p53 wt-3′UTRForward: ′-CG***ACGCGT***CCTGATACAGATGCTACTTGA-3′; H p53 wt-3′UTRReverse: 5′-CAGGTGGCAGCAAAGTTT***AGATCT***TCC-3′; H p53 mut-3′UTR Forward: 5′- CTGACAGCCTCCCACCCCCATC-3′; H p53 Mut-3′UTR Reverse: 5 (- GTGGGGAACAAGAAGTGGAGA-3′.

### Dual luciferase reporter assay

The reporter plasmids containing wt or mutated 3′UTRs and control plasmid [cytomegalovirus (CMV)–driven renilla luciferase construct, pRL-CMV] (Promega), and miRNA mimics or negative control mimics were co-transfected into cells using Lipofectamine 2000 (Invitrogen), according to the manufacturer’s instructions. After 48 h, reporter activity was measured using a dual luciferase reporter gene assay kit (Promega).

### Quantitative real-time PCR

Total RNA was isolated using the TRIZOL reagent (Invitrogen). Relative mRNA and pri-miRNA levels were determined by qRT-PCR using a Stratagene MX3000P system, One-Step SYBR PrimeScript RT-PCR Kit (Takara), and specific primers. Cycle threshold (CT) values were determined using MX3000p software (version 4.10) with amplification-based threshold determination and adaptive baseline analysis options. GAPDH was used as the endogenous control.

MiRNAs were quantified using the All-in-One miRNA qRT-PCR Detection Kit (GeneCopoeia, Rockville, MD, USA). For single-step cDNA synthesis, poly(A) polymerase was used to add poly(A) tails to the 3′ end of miRNAs, and M-MLV reverse transcriptase and a unique oligo(dT) adaptor primer were used to reverse transcribe poly(A) miRNAs. An All-in-One qPCR Mix containing SYBR Green was used to detect specific reverse-transcribed miRNAs using a Universal Adaptor PCR primer and a miRNA-specific primer. MiRNAs were normalized against U6 small nuclear RNA or RNU44 small Nucleolar RNA. The results were expressed in arbitrary units and were representative of three independent experiments. Primers for quantitative real-time PCR sequences are shown in Table S2.

### Statistical analysis

All data were processed using SPSS10.0 software, expressed as the mean ± SD and subjected to a Shapiro-Wilk (W) test for normality. For non-normally distributed data, the nonparametric Wilcoxon signed rank test was used to evaluate the statistical differences between groups. When the data were approximately normally distributed, the comparison of multiple group data was performed using one-way ANOVA, and multiple comparisons of means were analysed by the LSD method. Groups with values of P ≤ 0.05 were considered to be statistically significant.

## Additional Information

**How to cite this article**: Li, J. *et al*. Species-specific mutual regulation of p53 and miR-138 between human, rat and mouse. *Sci. Rep.* **6**, 26187; doi: 10.1038/srep26187 (2016).

## Supplementary Material

Supplementary Information
