# Supplementary material for: Species-specific mutual regulation of p53 and miR-138 between human, rat and mouse
Source: Sci Rep. 2016 May 17;6:26187. doi: 10.1038/srep26187 (PMC4869026; doi:10.1038/srep26187)
Supplement: Supplementary Information [file srep26187-s1.doc]

**Supplemental Information**

**Species-specific mutual regulation of p53 and miR-138 between human, rat and mouse**

Jie Li^1#^ , Wei Xia^1#^ , Xueting Su^1^, Xingliang Qin^1^, Ying Chen^1^, Shaohua Li^1^, Jie Dong^1^, Hongmei Ding^1^, Hui Li^1^, Aixue Huang^1^, Xingfeng Ge^1^, Lvbin Hou^1^, Chaonan Wang^1^, Leqiao Sun^1^, Chenjun Bai^1^, Xuelian Shen^1^, Tao Fang^1^, Yuanlin Liu^2^, Yi Zhang^2^, Hongru Zhang^3^, Hongwen Zhang^4^*, Ningsheng Shao^1^*

^1^Department of Biochemistry and Molecular Biology, Institute of Basic Medical Sciences, Beijing 100850, China

^2^Department of Cell Biology, Institute of Basic Medical Sciences, Beijing 100850, China

^3^Otorhinolaryngological department, Haidian Section of Peking University third Hospital

^4^Department of Interventional Radiology, General Hospital of Fuzhou, Fuzhou, China

* To whom correspondence should be addressed.

Tel/Fax: +8610-68213039, Email: shaonsh@hotmail.com (N-S.Shao); Tel: +86-591-22859125, Email: zhanghw1983@126.com (H-W, Zhang).

^#^ The authors contributed equally to this paper.

**Supplemental Figure**


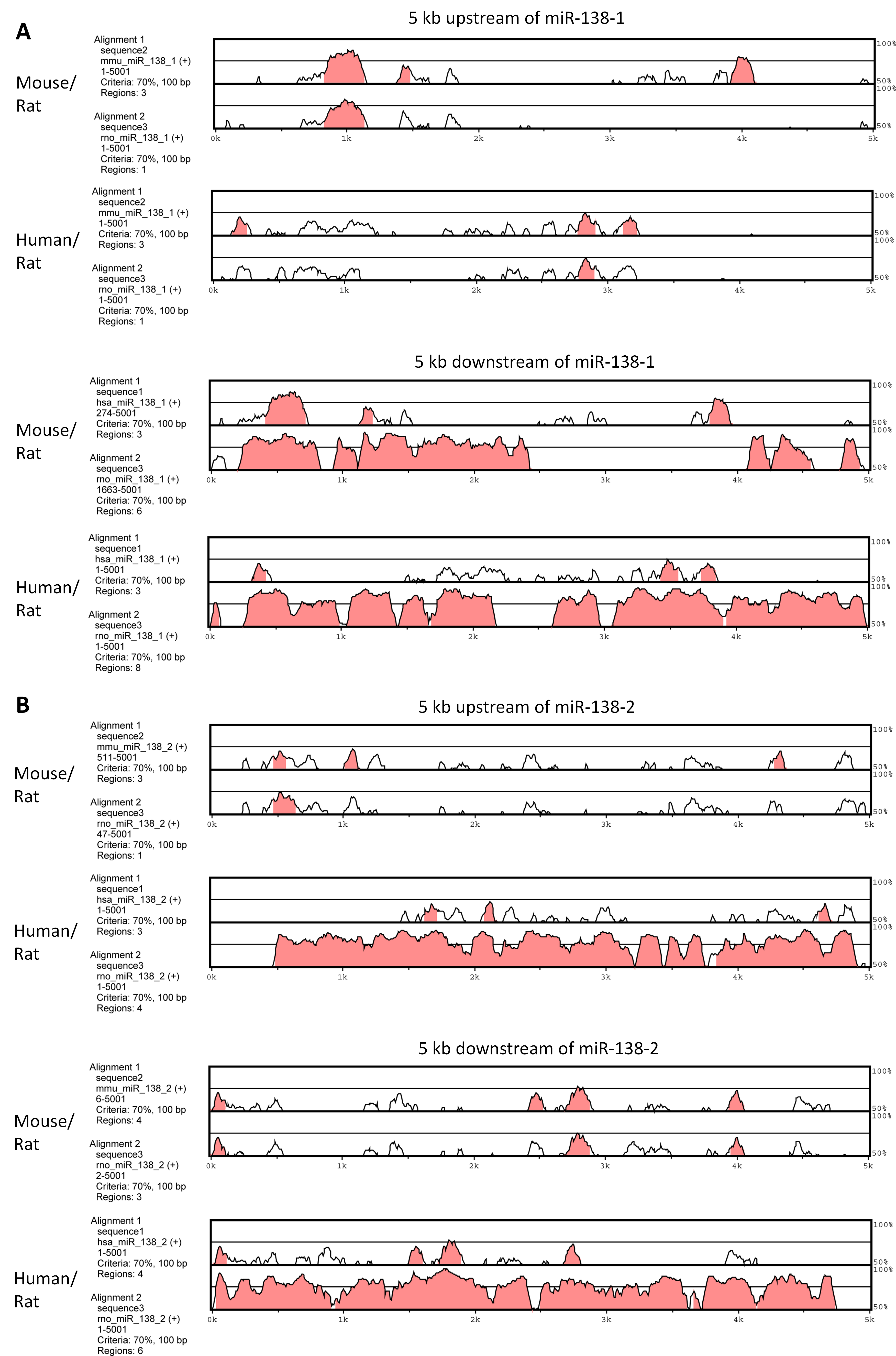


Figure S1. Cross-species Vista alignment of the 5kb upstream or downstream of the miR-138 transcript (http://genome.lbl.gov/vista/mvista/submit.shtml). Peaks show the degree of homology. (A) Vista alignment of the 5kb upstream or downstream of the miR-138-1 transcript. (B) Vista alignment of the 5kb upstream or downstream of the miR-138-2 transcript.


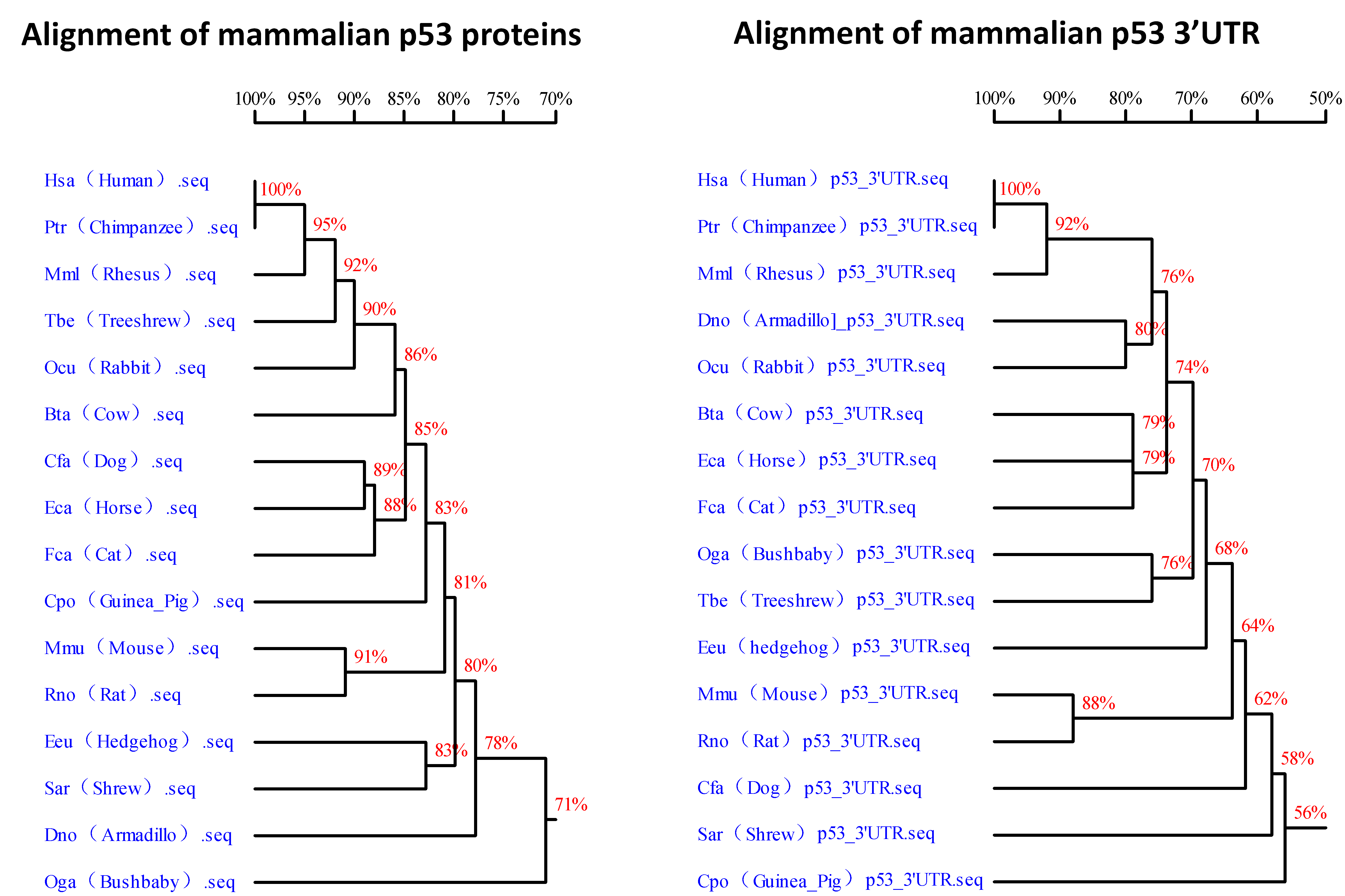


Figure S2. Alignment of mammalian p53 amino acid sequences and p53 3′ UTR nucleotide sequences.

**
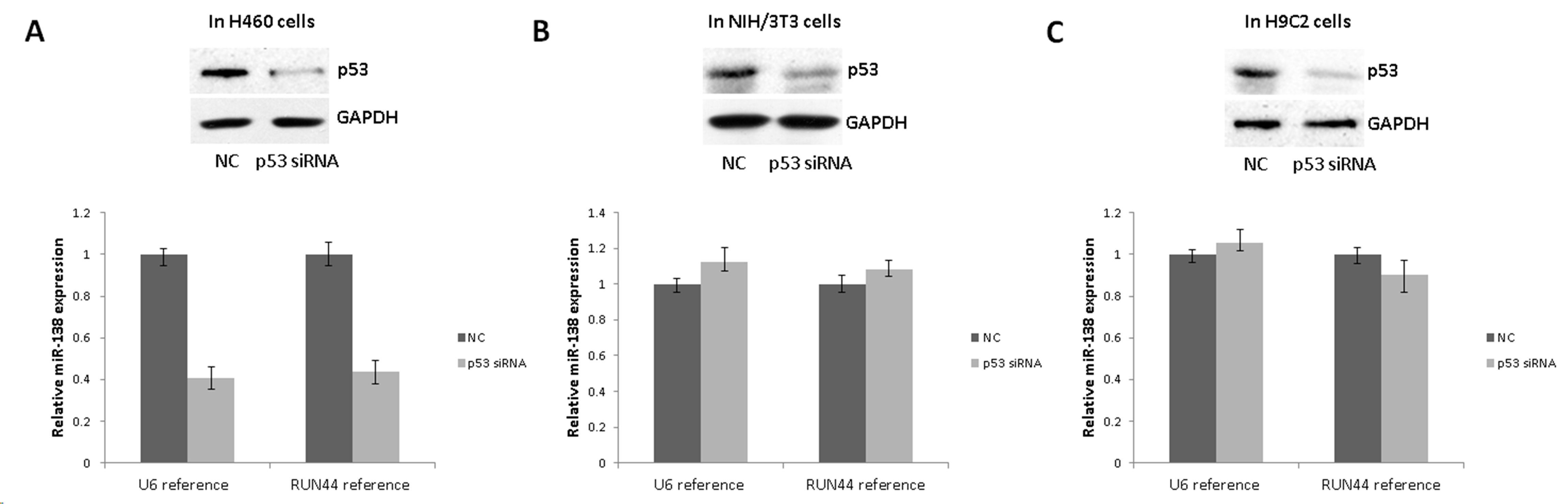
**

Figure S3. Compare miR-138 levels when U6 or RNU44 used as internal control (A, B and C) Western blot analyses were performed to examine the effects of p53 siRNA on endogenous p53 protein levels in H460,NIH/3T3 and H9C2 cells (top); quantitative RT-PCR assays were performed to examine miR-138 levels in three cell types (H460, NIH/3T3 and H9C2) treated with p53 siRNA for 48 h. MiRNAs were normalized against U6 small nuclear RNA or RNU44 small Nucleolar RNA respectively.

**Supplemental Tables**

Table S1. Primers for ChIP analysis

| Specie | Name | No. | Forward primer（5'-3') | Reverse primer(5'-3') |
| --- | --- | --- | --- | --- |
| Human | hsa-miR-138-1 | A | TTCACTTAGCGTATTCAGC | CTCCTACTCTGGCACCTA |
|  | hsa-miR-138-1 | B | GTAAGTAGTAATGGAAGGACAC | ATCTGAACCTGACTGGAAG |
|  | hsa-miR-138-2 | A | ACCGTCTCTTCCATTAGAAT | TTCCACTGCGTTTATTGAG |
| Mouse | mmu-miR-138-1 | A | GACACGGACATAAGACACA | GTCGAAGATGACTTACAATGG |
|  | mmu-miR-138-1 | B | GCTACAGATGGTCTCCTTAT | TGACACATGAGCACACTC |
|  | mmu-miR-138-1 | C | CAGCCGCTTGTTCATCTT | GAGGACATTAGAAATTGGGAAG |
|  | mmu-miR-138-1 | D | GCCTGACTTGTATATTCTGA | ACCTTGTTCTGAGACTGAG |
|  | mmu-miR-138-1 | E | GGTGGAGGAGAAGAATGTAG | AGGCTGTTGCTCTAGGTT |
|  | mmu-miR-138-2 |  | - | - |
| Rat | rno-miR-138-1 | A | CTGACTCCATCCTCTGACA | AACCACTAGCCAACACAAG |
|  | rno-miR-138-1 | B | CTGTATCATAACTTCTGTGAGG | GTGTGCGTGTGTCCATAT |
|  | rno-miR-138-2 |  | - | - |
|  | hTERT |  | CAGGCCGGGCTCCCAGTGGA | CGTGCGCTCCCTGCTG |
|  | Negative control |  | GAAGAACGGCATCAAGGTG | CTGGGTGCTCAGGTAGTGG |

Table S2. Primers for quantitative real-time PCR

| Name | Sequence（5'-3') | |
| --- | --- | --- |
| Human p53 | Forward | GAGGTTGGCTCTGACTGTACC |
|  | Reverse | TCCGTCCCAGTAGATTACCAC |
| Mouse p53 | Forward | GTCACAGCACATGACGGAGG |
|  | Reverse | TCTTCCAGATGCTCGGGATAC |
| Rat p53 | Forward | ACAGCGTGGTGGTACCGTAT |
|  | Reverse | GGAGCTGTTGCACATGTACT |
| GAPDH | Forward | ACAACTTTGGTATCGTGGAAGG |
|  | Reverse | GCCATCACGCCACAGTTTC |
| pri-mR-138-1 | Forward | TCTGTCCTCTCTCCACCT |
|  | Reverse | GAAGTAGCCGTTCTCTGATT |
| pri-miR-138-2 | Forward | AGTTCTGGTATCGTTGCT |
|  | Reverse | AGGATGGGTATGATGCAA |
| U6 | Forward | CTCGCTTCGGCAGCACA |
|  | Reverse | GCGAGCACAGAATTAATACGAC |
| RNU44 | Forward | CCTGGATGATGATAGCAAATGC |
|  | Reverse | GAGCTAATTAAGACCTTCATGTT |
| miR-138 | Forward | GCCGCAGCTGGTGTTGTGAAT |
|  | Reverse | GCGAGCACAGAATTAATACGAC |
| Unique oligo(dT) adaptor primer |  | GCGAGCACAGAATTAATACGACTCACTATAGGTTTTTTTTTTTTTTTTTTVN |
